# Supplementary material for: Genomic and Phenotypic Heterogeneity of Clinical Isolates of the Human Pathogens Aspergillus fumigatus, Aspergillus lentulus, and Aspergillus fumigatiaffinis
Source: Front Genet. 2020 May 12;11:459. doi: 10.3389/fgene.2020.00459 (PMC7236307; doi:10.3389/fgene.2020.00459)
Supplement: Supplementary file 2 [file Image_1.PDF]

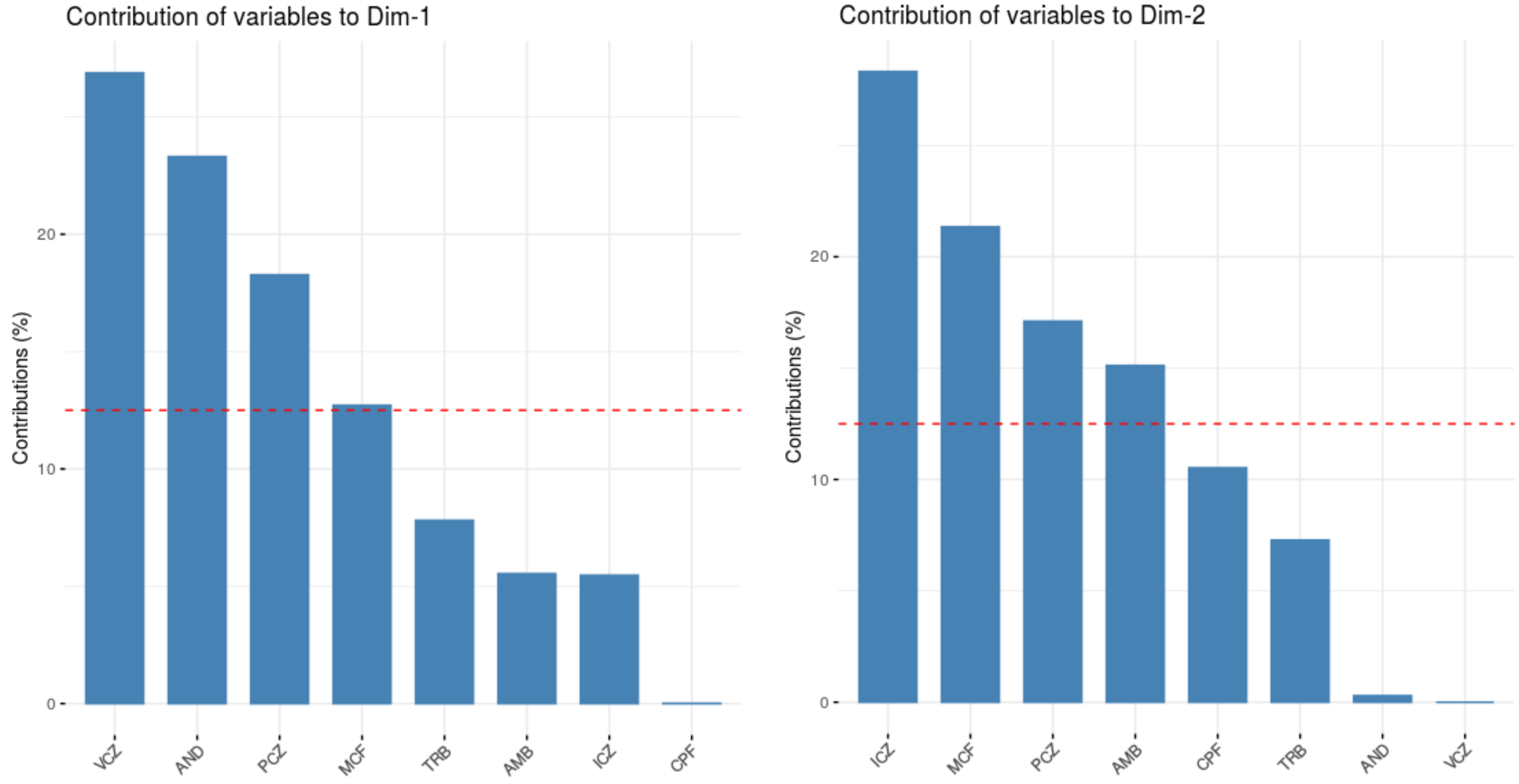

Supplementary Figure 1. Contribution of variables (drug MIC/MEC) to principal components.
