# Supplementary material for: Genomic and Phenotypic Heterogeneity of Clinical Isolates of the Human Pathogens Aspergillus fumigatus, Aspergillus lentulus, and Aspergillus fumigatiaffinis
Source: Front Genet. 2020 May 12;11:459. doi: 10.3389/fgene.2020.00459 (PMC7236307; doi:10.3389/fgene.2020.00459)
Supplement: Supplementary file 4 [file Image_3.PDF]

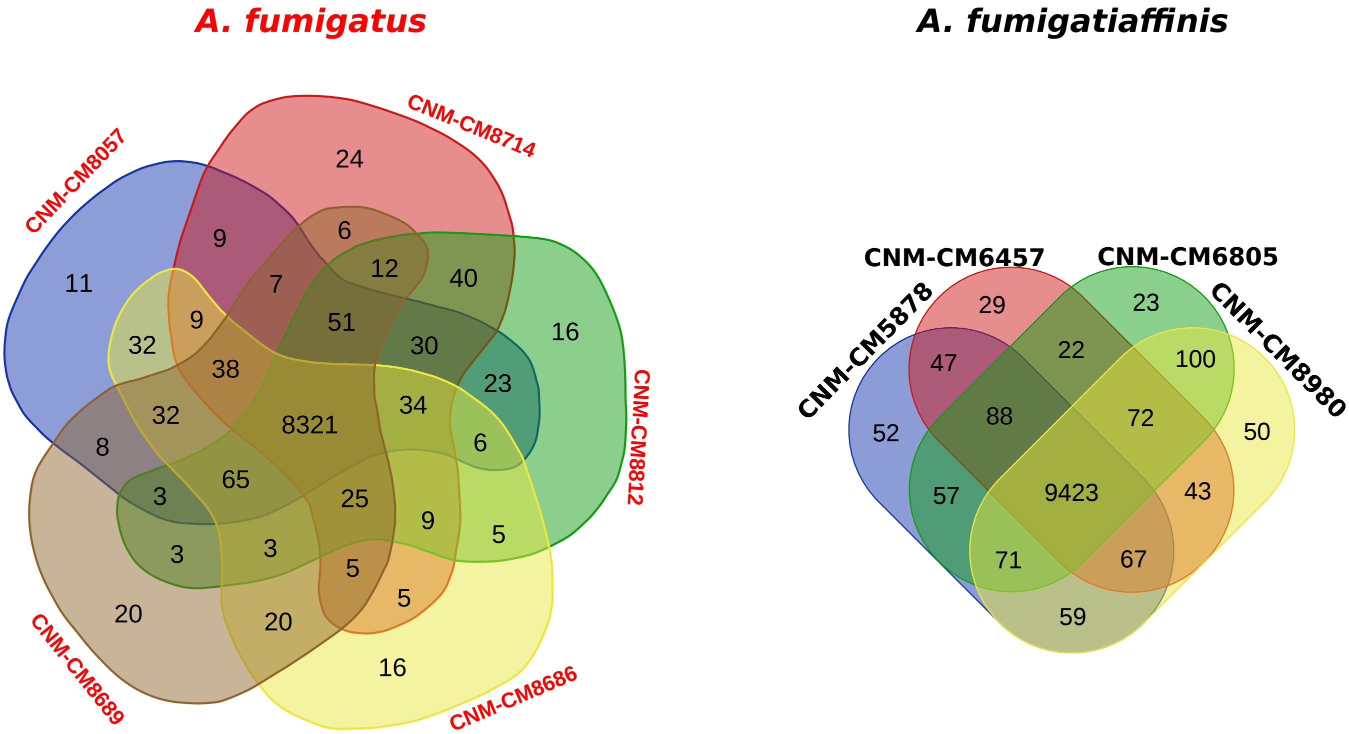

Supplementary figure 3. Venn Diagram of orthogroups shared by strains in each species for *A. fumigatus* and *A. fumigatiaffinis*.
